# Migration risk factors and their impact on psychological distress among Unaccompanied Migrant Minors in Spain

**DOI:** 10.1192/j.eurpsy.2025.1660

**Published:** 2025-08-26

**Authors:** P. Cristóbal Narváez, M. Franch-Roca, R. El Hafi, I. Giné, L. Aparicio, M. Febas, H. Sainz Elias, Y. Osorio, J. M. Haro

**Affiliations:** 1 Research, Innovation and Teaching Unit, Parc Sanitari Sant Joan de Déu, Barcelona; 2 Centre for Biomedical Research on Mental Health (CIBERSAM), Madrid; 3 Servicio de Atención a la Migración en Salud Mental (SATMI), Parc Sanitari Sant Joan de Déu, Barcelona; 4 Children and Youth Integration Area, Sant Joan de Déu Terres de Lleida, Lleida, Spain

## Abstract

**Introduction:**

Unaccompanied Migrant Minors (UMMs) who travel alone and live apart from their families are particularly vulnerable to mental health issues and social exclusion in Spain. Risk factors related to the migratory cycle, including travelling alone, living away from family, and experiencing discrimination, can negatively impact their mental health and increase the risk of social exclusion.

**Objectives:**

This study aims to describe the profile of newly arrived UMM and identify the relevant health risk factors among them, considering factors before, during, and after migration and their impact on psychological distress.

**Methods:**

The study involved face-to-face interviews with 230 minors in foster care placements. The interviews covered sociodemographic information, education and employment situations, factors related to the migratory process (before, during, and after migration), health status, and psychological distress. They were conducted in Arabic or French and translated into Spanish.

**Results:**

The findings revealed that UMMs generally perceived themselves as having good health before migration. However, they often held unrealistic expectations about their new life. Upon arrival, they had to cope with post-migration stressors such as stress (β = 0.468, SE = 0.142, p = 0.001) and discrimination (β = 0.357, SE = 0.121, p = 0.003), which adversely affected their mental health.

**Conclusions:**

The study highlights the impact of post-migration factors on psychological distress among newly arrived UMM. It underscores the need for comprehensive mental health care that considers the different stages of the migratory cycle. Additionally, it advocates for promoting cross-cultural mental health care models and developing policies and services to address and mitigate the effects of post-migration factors, including discrimination against UMMs in Spain.

**Disclosure of Interest:**

None Declared

